# Manejo percutáneo de hemoptisis severa en paciente posoperada de cirugía de fontan

**DOI:** 10.47487/apcyccv.v1i3.74

**Published:** 2020-09-30

**Authors:** Katherine Alcalá-Marcos, Giorgio Gabino-González, Abraham Flores-Gamero, César Conde Vela

**Affiliations:** 1 Servicio de Cardiología Perioperatoria - Instituto Nacional Cardiovascular - INCOR EsSalud. Lima, Perú Servicio de Cardiología Perioperatoria - Instituto Nacional Cardiovascular - INCOR EsSalud Lima Perú; 2 Servicio de Cardiología Clínica - Instituto Nacional Cardiovascular - INCOR EsSalud. Lima, Perú Servicio de Cardiología Clínica - Instituto Nacional Cardiovascular - INCOR EsSalud Lima Perú; 3 Servicio de Cardiología Invasiva - Instituto Nacional Cardiovascular - INCOR EsSalud. Lima, Perú Lima Perú

**Keywords:** Procedimiento de Fontan, Hemoptisis, Fístula, Fontan Procedure, Hemoptysis, Fistula

## Abstract

Se presenta el caso de una paciente con antecedente de ventrículo único por atresia tricuspídea, doble vía de salida del ventrículo izquierdo e hipoplasia del anillo de la arteria pulmonar. La paciente fue tratada con cirugía de Glenn a los 7 años de vida y, a los 16, se le realizó cirugía de Fontan extracardíaco fenestrado. Luego de 1 mes de realizada la cirugía presentó hemoptisis severa sin adecuada respuesta al manejo médico. Se realizó cierre percutáneo de las fístulas aortopulmonares con *coils*, con evolución favorable, sin presentar recurrencias de hemoptisis.

## Abstract (en)

We present the case of a patient with a history of single ventricle, tricuspid atresia, double outlet of the left ventricle and hypoplasia of the pulmonary artery ring. The patient was treated with Glenn’s surgery at the age of 7 and at age 16, the patient received fenestrated extracardiac Fontan surgery. After 1 month of Fontan surgery she presented severe hemoptysis without adequate response to medical management. Percutaneous closure of aortopulmonary fistulas with coils was performed, with favorable evolution, without presenting recurrences of hemoptysis.

El procedimiento de Fontan constituye una excelente terapia paliativa en casos de circulación univentricular, en estos pacientes se suele observar el desarrollo de múltiples fístulas aortopulmonares que pueden condicionar diversas complicaciones. La hemoptisis relacionada al sangrado de las colaterales aortopulmonares constituye un cuadro poco frecuente, pero puede llegar a ser fatal ^1^. Presentamos el caso de una paciente que desarrolló hemoptisis severa secundaria a colaterales aortopulmonares posterior a cirugía de Fontan, la cual se manejó mediante una estrategia de intervencionismo con evolución favorable.

## Descripción del caso

**Figura 1:**
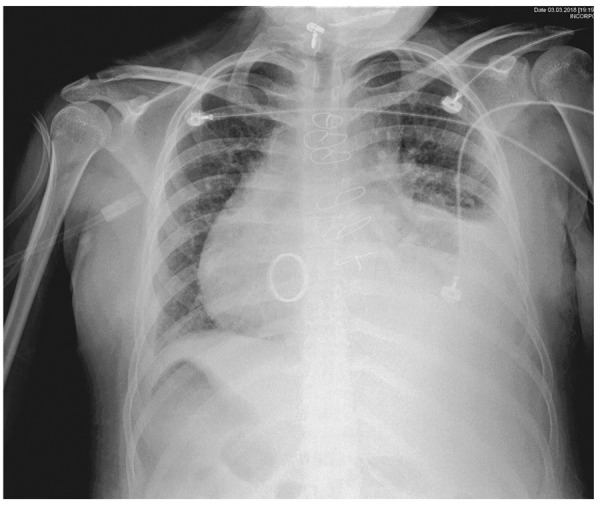
Radiografía de tórax mostrando efusión pleural izquierda moderada.

Paciente mujer de 16 años con antecedente de *situs inversus* en dextrocardia, ventrículo único por atresia tricuspídea, doble vía de salida del ventrículo izquierdo e hipoplasia del anillo de la arteria pulmonar. A los 7 años tuvo cirugía de Glenn con preservación del flujo anterógrado ventrículo-pulmonar. A los 16 años de edad se realizó cirugía de Fontan extracardíaco fenestrado (anastomosis de la vena cava inferior a la rama izquierda de la arteria pulmonar) y cambio de válvula auriculoventricular sistémica por una prótesis mecánica por insuficiencia severa. Fue dada de alta sin intercurrencias, manteniendo saturación entre 85-91%, y en tratamiento con ácido acetilsalicílico y warfarina. Un mes después del alta presentó tos persistente asociada con esputo hemoptoico que se incrementó en frecuencia progresando a hemoptisis franca en el transcurso de 3 días por lo que ingresa a emergencia. Al ingreso estuvo estable hemodinámicamente, afebril, y con disnea y tos intermitente. Al examen físico se encontró frecuencia cardíaca 90 lpm, presión arterial 110/60 mmHg, frecuencia respiratoria 22 rpm, saturación de oxigeno de 86% con oxígeno ambiental, disminución del murmullo vesicular en tercio inferior de hemitórax izquierdo, no crepitantes. Los exámenes de laboratorio demostraron hemoglobina de 13 g/dL, INR 2,5. Se revirtió la anticoagulación con plasma fresco congelado y se administró ácido tranexámico. En la radiografía de tórax se observó velamiento de la mitad inferior del hemitórax izquierdo compatible con efusión pleural **(**Figura 1**)**. Se colocó un tubo de drenaje obteniéndose 800 mL de líquido seroso. Los estudios microbiológicos en esputo para *Mycobacterium tuberculosis* resultaron negativos.

En la ecocardiografía transtorácica se constató que la prótesis valvular mecánica sistémica se encontraba normofuncionante; la función sistólica del ventrículo único se mantenía preservada al igual que el flujo anterógrado a través de la arteria pulmonar. La angiotomografía cardíaca mostró permeabilidad del conducto Fontan extracardíaco, hipoplasia de anillo pulmonar, flujo anterógrado pulmonar con ramas pulmonares dilatadas y múltiples colaterales aortopulmonares, la mayor de 2,5 mm de diámetro. Durante el segundo día de observación la paciente presentó nuevos episodios de hemoptisis de hasta 500 mL por lo que pasó a la unidad de cuidados intensivos cardiológicos. Ante una paciente con hemoptisis de grado severo, que requiere anticoagulación por ser portadora de prótesis sistémica mecánica, se decidió el cierre percutáneo de las colaterales aortopulmonares y la oclusión del flujo anterógrado del tronco de la arteria pulmonar.

**Figura 2:**
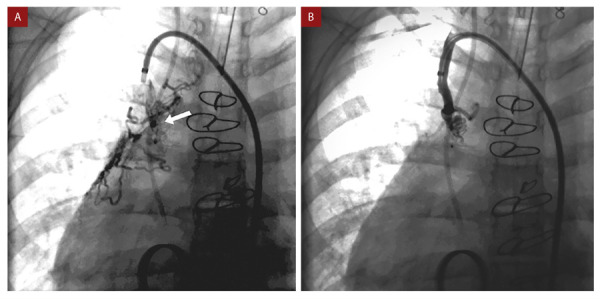
Se observa colateral aortopulmonar proveniente de arteria subclavia derecha antes (flecha blanca) **(A)** y después de implante del *coil* **(B)**.

El cateterismo cardíaco se realizó bajo anestesia general, la presión del sistema Fontan se encontró incrementada (19 mmHg) respecto al estudio previo a la cirugía de Fontán en la AP (presión en arteria pulmonar 15 mmHg). Se constató flujo anterógrado desde tronco de la arteria pulmonar hacia las ramas pulmonares y se identificaron múltiples colaterales aortopulmonares, las mayores originándose de las arterias subclavias. Se implantó un *stent* cubierto de 4,5 cm en la bifurcación de las ramas de la arteria pulmonar con el objetivo de eliminar el flujo anterógrado desde el tronco de la arteria pulmonar, evidenciándose en el control angiográfico una disminución importante de este flujo. Se realizó la oclusión con *coils* de las colaterales aortopulmonares más importantes de las arterias subclavias izquierda y derecha **(**Figura 2**y**3**)**. Posteriormente, la paciente se mantuvo estable, no presentó nuevos episodios de hemoptisis, permitiendo el reinicio de la anticoagulación. Fue dada de alta a los 10 días. En el seguimiento a los 6 meses, en la angiotomografía cardíaca se constató la presencia del *stent* cubierto en la bifurcación de las arterias pulmonares, la permeabilidad del sistema Fontan y las colaterales aortopulmonares ocluidas con *coils* **(**Figura 4**)**. Un año después, la ecocardiografía de control mostró función sistólica ventricular preservada, prótesis mecánica sistémica normofuncionante, sistema Fontan permeable, flujo anterógrado pulmonar mínimo. La paciente mantiene clase funcional II, con saturación arterial entre 89-93% y no se reportó recurrencia de la hemoptisis.

**Figura 3:**
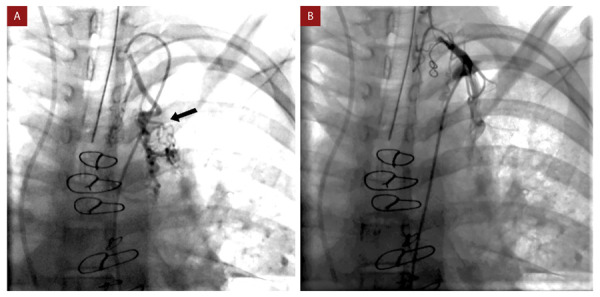
Imagen de colateral aortopulmonar proveniente de arteria subclavia izquierda (flecha negra) **(A)**. Oclusión de colateral luego de implante de *coil* **(B)**.

**Figura 4:**
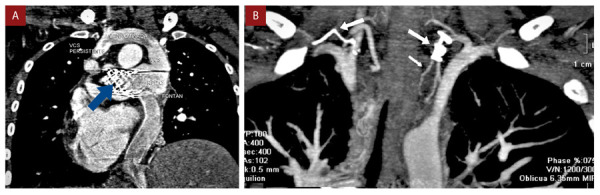
Control angiotomográfico luego de 6 meses del procedimiento. *Stent* recubierto en origen de arterias pulmonares **(A) Flecha azul**. Presencia de *coils* a nivel de colaterales aortopulmonares (flechas blancas) **(B)**.

## Discusión

La hemoptisis es una complicación poco frecuente, pero de gran importancia en pacientes operados de Fontan, llegando en ciertos casos a ser fatal ^1^. En un estudio publicado en 2014 se realizó el seguimiento de 412 pacientes operados de Fontan durante un período de 11 años, y la hemoptisis se presentó en 3,1%, representando el 70,5% de las complicaciones hemorrágicas ^1^. La hemoptisis en pacientes con cardiopatía congénita cianótica puede originarse debido a colaterales aortopulmonares o a malformaciones arteriovenosas ^1^^-^^5^. Las colaterales aortopulmonares que se encuentran en relación cercana al árbol bronquial pueden dilatarse, protruir hacia la mucosa bronquial y romperse generando hemoptisis importante ^5^. Esto puede desencadenarse de forma espontánea, ya sea por un incremento de flujo progresivo hacia las colaterales aortopulmonares, o por alguna lesión aguda o crónica de las vías aéreas, como procesos inflamatorios infecciosos o no infecciosos tales como reflujo gastroesofágico o alergias ^1^^,^^2^^,^^5^.

La presencia de colaterales aortopulmonares suele ser frecuente en pacientes operados de Fontan con una prevalencia de hasta 30%, originándose en su mayoría de las arterias mamarias internas, del tronco tirocervical y de las arterias intercostales ^2^; suele ser mayor en aquellos con cardiopatía congénita cianótica cuya cirugía de Fontan se realizó a mayor edad, ya que tiempos de hipoxia más prolongados incrementarían la actividad angiogénica pulmonar ^1^.

La recurrencia de la hemoptisis suele ser muy frecuente con manejo médico, alcanzando el 60% ^3^^,^^5^, e incluso mayor en casos de mantener terapia anticoagulante como en el caso de nuestra paciente, que la necesitaba por ser portadora de prótesis mecánica. El impacto de la hemoptisis va en proporción a su severidad, generando en muchos casos necesidad de ventilación mecánica o múltiples transfusiones; alcanza una mortalidad de 22% en casos severos y de 71% en hemoptisis masiva ^4^^-^^6^.

El tratamiento definitivo va dirigido a la causa de la hemoptisis. En el presente caso se identificó mediante la angiotomografía de tórax la presencia de múltiples colaterales aortopulmonares. El manejo percutáneo de las colaterales consiste en su oclusión por medio de *coils* o a través de embolización ^2^^,^^5^^,^^6^. En la mayoría de los casos suele haber múltiples colaterales aortopulmonares, realizándose el cierre en aquellas de mayor tamaño, mientras que se puede optar por un manejo conservador con las de diámetro reducido ^2^^,^^6^. El tratamiento percutáneo se considera como la terapia de elección, con una tasa de éxito de hasta 80%, para control del episodio agudo, además de disminuir la recurrencia de hemoptisis; asimismo, se asocia a una menor tasa de complicaciones durante el procedimiento (0,1%) ^5^^-^^11^.

La persistencia de flujo anterógrado ventrículo-pulmonar puede generar una sobrecarga de volumen ventricular, así como el aumento de presiones pulmonares y del sistema Fontan, asociándose con efusión pleural persistente y disfunción ventricular ^12^^,^^13^. El cierre del flujo anterógrado podría disminuir la sobrecarga de presiones en el sistema Fontan y reducir los eventos de hemoptisis al disminuir la formación de colaterales aortopulmonares ^13^. En nuestra paciente, a través del cierre percutáneo de las colaterales aortopulmonares de mayor tamaño y con la disminución del flujo anterógrado se consiguió detener el episodio agudo de sangrado, sin presentarse mayores complicaciones durante el procedimiento y mostrando en el seguimiento una adecuada evolución sin recurrencias de hemoptisis al año.

## Conclusión

Se presenta un caso poco frecuente de hemoptisis severa tras cirugía de Fontan, causada por el sangrado a partir de colaterales aortopulmonares, que no tuvo respuesta adecuada a la terapia médica. Se realizó el cierre percutáneo de las colaterales aortopulmonares y la oclusión del flujo anterógrado pulmonar, consiguiendo mejoría clínica y evitando la aparición de nuevos episodios de hemoptisis.
